# H_2_S and homocysteine control a novel feedback regulation of cystathionine beta synthase and cystathionine gamma lyase in cardiomyocytes

**DOI:** 10.1038/s41598-017-03776-9

**Published:** 2017-06-16

**Authors:** Shyam Sundar Nandi, Paras Kumar Mishra

**Affiliations:** 1 Department of Cellular and Integrative Physiology, University of Nebraska Medical Center, Omaha, NE 68198 USA; 2 Department of Anesthesiology, University of Nebraska Medical Center, Omaha, NE 68198 USA

## Abstract

Hydrogen sulfide (H_2_S), a cardioprotective gas, is endogenously produced from homocysteine by cystathionine beta synthase (CBS) and cystathionine gamma lyase (CSE) enzymes. However, effect of H_2_S or homocysteine on CBS and CSE expression, and cross-talk between CBS and CSE are unclear. We hypothesize that homocysteine and H_2_S regulate CBS and CSE expressions in a dose dependent manner in cardiomyocytes, and CBS deficiency induces cardiac CSE expression. To test the hypothesis, we treated murine atrial HL1 cardiomyocytes with increasing doses of homocysteine or Na_2_S/GYY4137, a H_2_S donor, and measured the levels of CBS and CSE. We found that homocysteine upregulates CSE but downregulates CBS whereas Na_2_S/GYY4137 downregulates CSE but upregulates CBS in a dose-dependent manner. Moreover, the Na_2_S-treatment downregulates specificity protein-1 (SP1), an inducer for CSE, and upregulates miR-133a that targets SP1 and inhibits cardiomyocytes hypertrophy. Conversely, in the homocysteine-treated cardiomyocytes, CBS and miR-133a were downregulated and hypertrophy was induced. *In vivo* studies using CBS+/−, a model for hyperhomocysteinemia, and sibling CBS+/+ control mice revealed that deficiency of CBS upregulates cardiac CSE, plausibly by inducing SP1. In conclusion, we revealed a novel mechanism for H_2_S-mediated regulation of homocysteine metabolism in cardiomyocytes, and a negative feedback regulation between CBS and CSE in the heart.

## Introduction

Elevated levels of homocysteine (Hcy), a non-protein coding amino acid, is associated with cardiovascular diseases (CVD)^1^ and mortality^2–4^. However, whether increased (above 15 µmol/L in plasma) levels of Hcy, referred to as hyperhomocysteinemia (HHcy), is a cause or an effect^5^ is debated because Hcy lowering interventions failed to reduce cardiovascular events and the rate of mortality in clinical trials^6, 7^. Hcy level is decreased in clinical trials by folic acid treatment, which remethylate Hcy to methionine. Another pathway to reduce Hcy level is transsulfuration of Hcy to hydrogen sulfide (H_2_S), where cystathionine beta-synthase (CBS) and cystathionine gamma lyase (CSE) enzymes play crucial roles^8^. These two enzymes are biosynthesized by different pathways, and their expressions are tissue specific^9, 10^. CBS is the dominant H_2_S-producing enzyme in the cardiovascular system^11^ and CSE/H_2_S pathway has a crucial role in cardiovascular disease^9^. The expression of CSE is regulated by the activity of specificity protein -1 (SP1)^12–14^. CBS and CSE endogenously produce H_2_S^15^, a cardioprotective gaseous molecule that regulates several signaling pathways associated with the mitigation of adverse cardiac remodeling^10, 16^. Downregulation of CBS and/or CSE enzyme decreases H_2_S production due to impaired transulfuration of Hcy that leads to HHcy. However, it is unclear whether increased Hcy accumulation or reduced H_2_S production is the main cause for pathological cardiac remodeling. In our previous study, we treated HL1 cardiomyocytes with Hcy and/or H_2_S donor Na_2_S, and measured cardiomyocytes hypertrophy. We found that Na_2_S mitigates Hcy-mediated cardiomyocyte hypertrophy by upregulating anti-hypertrophic miR-133a^17^. MiR-133a is a cardioprotective miRNA, which is downregulated in the failing heart of humans and mice^18, 19^. Although Na_2_S-mediated reduction of hypertrophy in HHcy cardiomyocytes indicates that reduced H_2_S production in HHcy cardiomyocytes may be a major cause for hypertrophy^17^, it is unclear whether Hcy or H_2_S regulates miR-133a levels in a dose-dependent manner. Moreover, the direct effect of HHcy or H_2_S on CBS and CSE enzymes is poorly understood. Further, whether a feedback regulation between CBS and CSE exist in the heart is unknown. In the present study, we used HL1 cardiomyocytes cell line, and CBS+/− (a model for HHcy) and sibling CBS+/+ mice to uncover the dose-dependent effects of Hcy or H_2_S on the levels of CBS and CSE in cardiomyocytes, and to investigate the feedback regulation between CBS and CSE in the heart.

## Results

### Dose-dependent effect of homocysteine or H_2_S donor Na_2_S on CBS and CSE in cardiomyocytes

**Figure 1:**
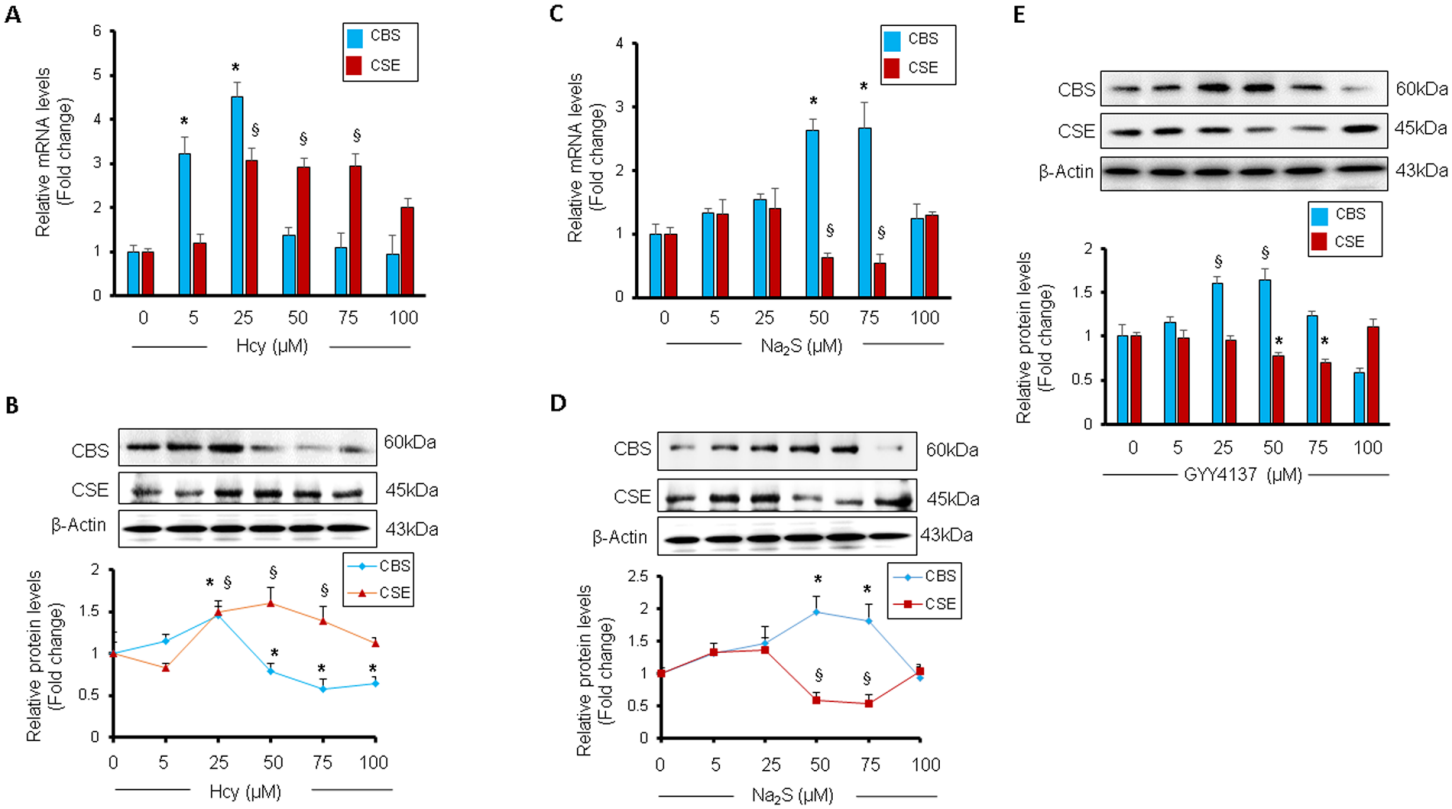
Dose-dependent effect of homocysteine or hydrogen sulfide donor Na_2_S or GYY4137 (H_2_S donor) on CBS and CSE levels in HL1 cardiomyocytes. (**A**) qPCR analyses of CBS and CSE after treatment with different dosages of homocysteine (Hcy), or (**C**) Na_2_S. (**B**) Western blot analyses of CBS and CSE after treatment with different doses of homocysteine (Hcy), or (**D**) Na_2_S. (**E**) Western blot analyses of CBS and CSE after treatment with different doses of GYY4137. Values are represented as mean ± SEM. “*” represents statistically significant value for CBS when compared to the untreated control, “§” represents statistically significant value for CSE when compared to the untreated control, and p < 0.05 is considered statistically significant. N = 3–5.

To determine the dose-dependent effects of Hcy or Na_2_S on CBS and CSE, we treated HL1 cardiomyocytes with 0, 5, 25, 50, 75, or 100 µM of Hcy or Na_2_S for 24 hours, and measured the mRNA and protein levels of CBS and CSE. We observed that up to 25 µM increasing doses Hcy elevated the mRNA and protein levels of both CBS and CSE as compared to the untreated cardiomyocytes (Fig. 1A,B). However, above 25 µM dose Hcy downregulated CBS but upregulated CSE (Fig. 1A,B), suggesting that HHcy induces CSE but suppresses CBS in cardiomyocytes. The effect of Na_2_S on the levels of CBS and CSE was different from Hcy. There was no difference at the mRNA and protein levels of CBS and CSE by increasing doses of Na_2_S up to 25 µM. However, 50 µM and 75 µM doses of Na_2_S increased the mRNA and protein levels of CBS but decreases the respective levels of CSE (Fig. 1C,D), suggesting that higher doses of Na_2_S induces CBS but inhibits CSE in cardiomyocytes. Notably, this trend was not maintained above 75 µM dose and at 100 µM dose Na_2_S did not change the levels of CBS and CSE (Fig. 1C,D). To corroborate the effect of H_2_S on CBS and CSE expression, we also treated HL1 cardiomyocytes with an alternate H_2_S donor, GYY4137, in the same dose-dependent manner. We observed that GYY4137 upregulated CBS at higher doses (25 µM and 50 µM) but downregulated CSE at higher doses (50 µM and 75 µM) (Fig. 1E), reinforcing that H_2_S has an opposite effect on CBS and CSE expressions in cardiomyocytes. Overall, we demonstrated that elevated levels of Hcy and H_2_S regulate CBS and CSE and they have an opposite effect on CBS and CSE expressions in cardiomyocytes (Fig. 1A–E).

### Mechanism for H_2_S-, and Hcy-mediated regulation of CSE in cardiomyocytes

**Figure 2:**
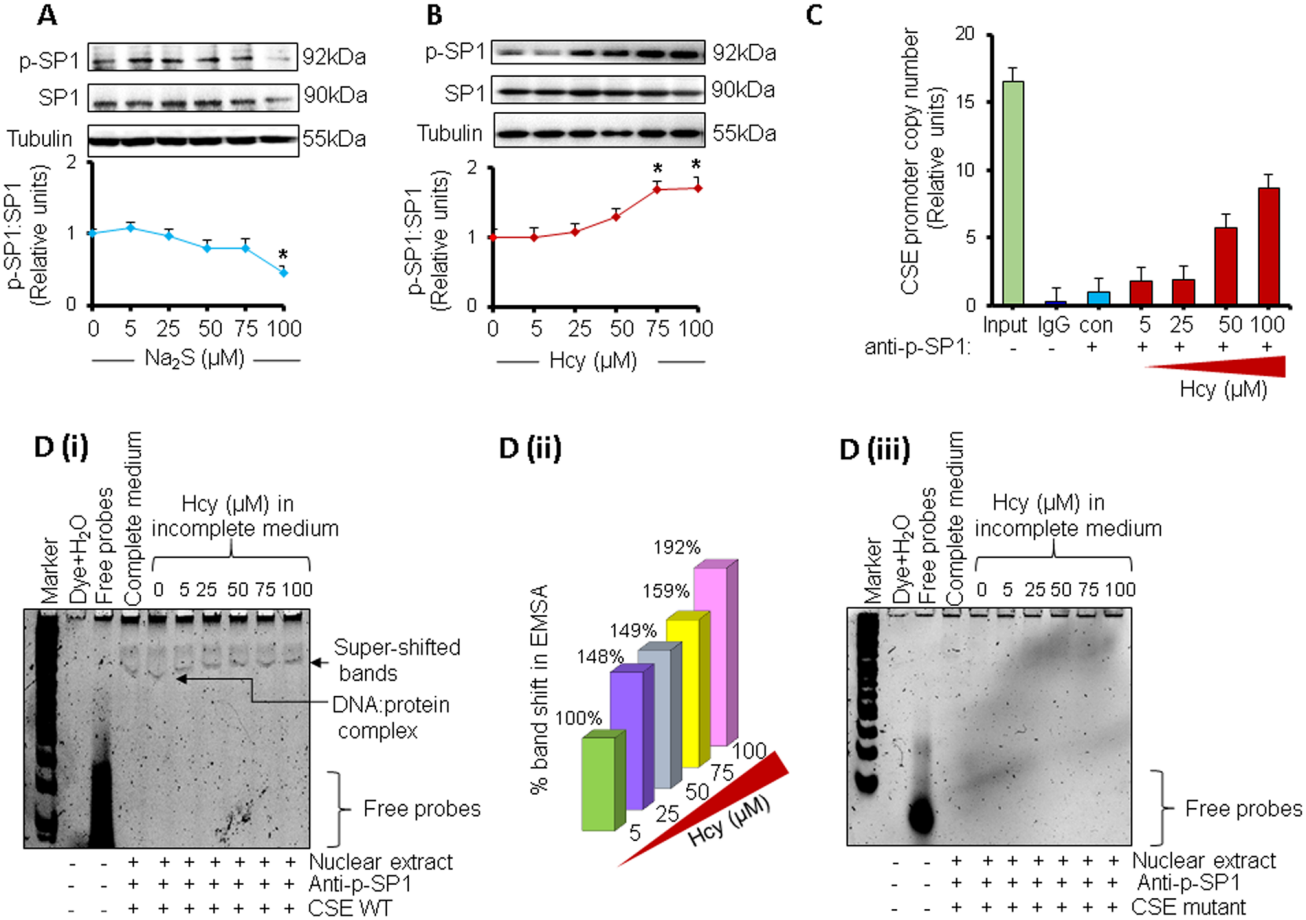
Effect of H_2_S or Hcy on SP1 activity in HL1 cardiomyocytes. (**A**) Western blot and densitometric analyses showing the activity of SP1 (p-SP1: SP1) in cardiomyocytes treated with different doses of Na_2_S or (**B**) Hcy. (**C**) Chromatin immunoprecipitation-qPCR assay showing amplification of CSE promoter copy number in cardiomyocytes treated with different doses of Hcy. (**D**) (**i**) Electrophoretic mobility shift assay showing WT CSE-SP-1 complex formation incubated with nuclear extracts from cardiomyocytes treated with different doses of Hcy. The super-shifted band represents binding of p-SP1 antibodies to the CSE-SP1 complex. (**D**) (**ii**) Quantification of percentage band shift in **‘D (i)’** in presence of p-SP1 antibody with different doses of Hcy. (**D**) (**iii**) Electrophoretic mobility shift assay (EMSA) showing no complex formation with mutant CSE-promoter. Values are represented as mean ± SEM. “*” represents statistically significant when compared to the untreated control. p < 0.05 is considered statistically significant. N = 5.

To determine the underlying molecular mechanism for H_2_S-mediated downregulation of CSE (Fig. 1D), we measured the activities of SP1, an inducer for CSE^12–14^, in HL1 cardiomyocytes treated with different doses of Na_2_S or Hcy. Our results demonstrated a trend of decreased SP1 activity at 25 µM dose of Na_2_S, which continued up to 75 µM dose but dramatically decreased at 100 µM dose (Fig. 2A). The decreased activity of SP1 at higher (50 µM and 75 µM) doses of Na_2_S (Fig. 2A) corresponds to the decreased levels of CSE (Fig. 1D) in HL1 cardiomyocytes, suggesting that Na_2_S downregulates CSE directly by suppressing the activity of SP1 in cardiomyocytes. Conversely, we found a trend of increased SP1 activity at 25 µM dose of Hcy, which continued up to 75 µM and 100 µM doses (Fig. 2B), showing that HHcy induces SP1. To validate the SP1 activity on CSE promoter, we performed Chromatin immunoprecipitation (ChIP) assay using p-SP1 specific antibody and CSE forward and reverse primers. Our results demonstrated that higher (50 µM and 75 µM) doses of Hcy increased the copy number of CSE promoter with respect to the untreated cells (Fig. 2C). It suggests that activated p-SP1 recruitment to the CSE promoter was increased at higher doses of Hcy, which results in an increased CSE transcriptional activity. The input chromatin was used as a positive control and normal rabbit IgG isotype was used as a negative control for ChIP assay (Fig. 2C). To corroborate the effect of Hcy on SP1 activity, we performed electrophoretic mobility shift assay (EMSA) using CSE promoter having normal SP1 consensus binding sites (CSE WT) and the mutant SP1 consensus binding sequence (CSE MUT). In CSE MUT, the SP1 consensus binding sequence on CSE promoter was scrambled. We observed that with increasing doses of Hcy, there was higher CSE WT- SP1 complex formation and enhanced gel-shift with p-SP1 antibody (Fig. 2D(i) and (ii)) but there was neither complex formation nor gel-shift in case of CSE MUT (Fig. 2D(iii)) suggesting that HHcy upregulates CSE by inducing SP1 binding to the CSE promoter in cardiomyocytes. Altogether, our findings provide a concrete evidence that Hcy upregulates whereas Na_2_S downregulates CSE directly by inducing and suppressing, respectively, the SP1 activity on CSE promoter in cardiomyocytes.

### MiR-133a targets CSE

**Figure 3:**
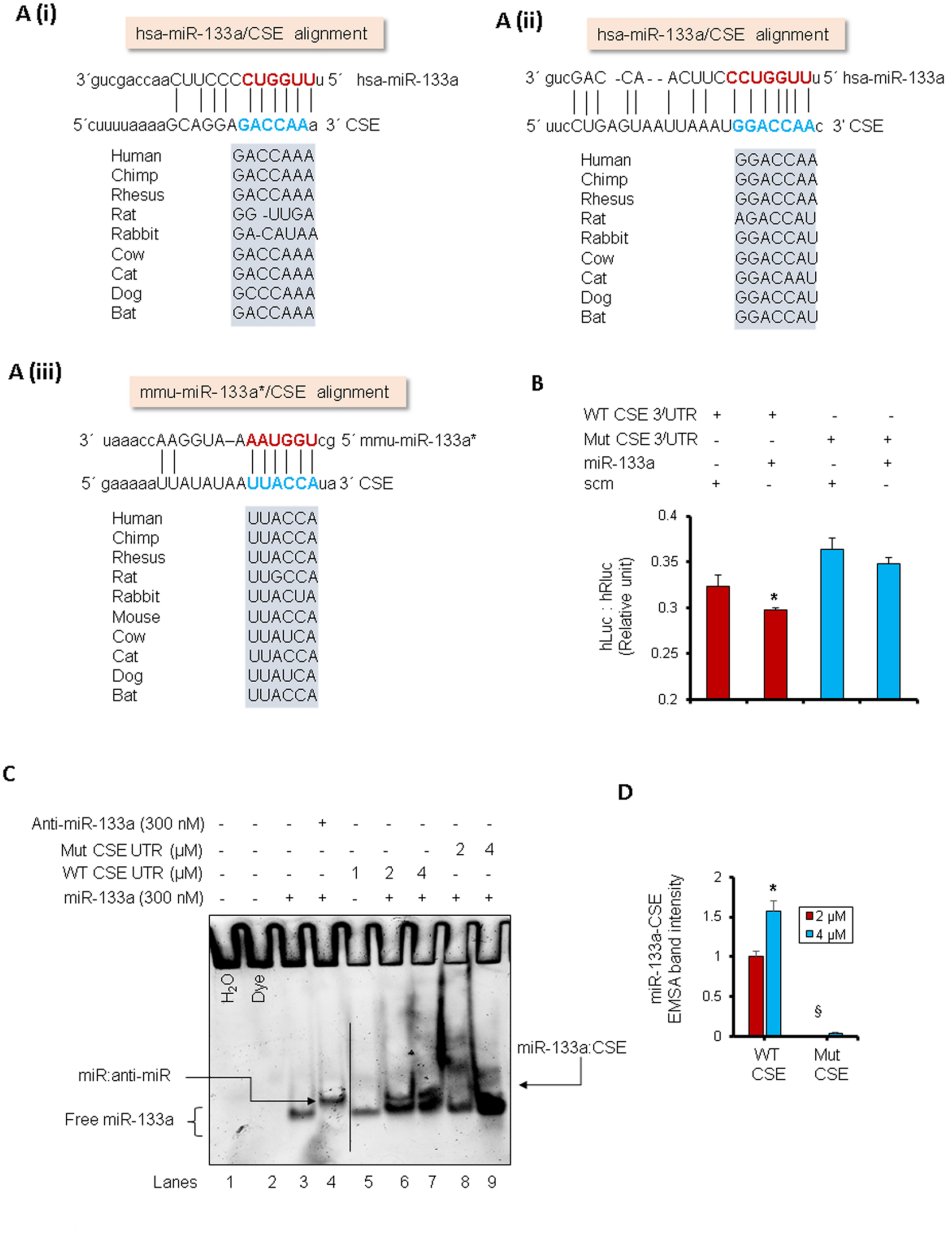
MiR-133a targets cystathionine gamma lyase (CSE). (**A**) (**i**–**iii**) Potential binding sites for miR-133a on 3′ UTR of CSE through in silico analysis. (**B**) Luciferase reporter assay showing the relative expression of luciferase activity (humanized luciferase, hLuc: humanized renilla luciferase, hRLuc) in miR-133a mimic-, or scrambled miRNA-treated HEK 293 cells co-transfected with either CSE 3′ UTR or mutant CSE 3′ UTR (miR-133a binding site is deleted in CSE 3′ UTR). hRLuc is an internal control for luciferase activity. Values are represented as mean ± SEM. p < 0.05 is considered statistically significant ( p = 0.04). N = 8. (**C**) miRNA-mRNA electrophoretic mobility shift assay showing binding of LNA miR-133a* probes with WT or mutant CSE 3′ UTR mRNA probes incubated with increasing concentration of 3′ UTRs (2 μM or 4 μM). (**D**) Quantification of miR-133a*-CSE band intensity in ‘**C**’ incubated with 2 μM or 4 μM of WT or mutant CSE promoter. Values are represented as mean ± SEM. p < 0.05 is considered statistically significant. N = 3. “*” represents statistically significant value for WT CSE at 4 μM when compared to 2 μM, “§” represents statistically significant value for mutant CSE when compared to the WT CSE at 2 μM.

In silico analyses showed that CSE is a potential target for miR-133a (Fig. 3A). We have reported that Na_2_S upregulates miR-133a in HHcy cardiomyocytes^17^. To determine whether miR-133a targets CSE, we performed luciferase reporter assay using normal 3′ untranslated region (WT CSE UTR) and mutant 3′UTR (Mut CSE UTR) of CSE. In Mut CSE UTR, the miR-133a binding sequence on 3′UTR of CSE was deleted. The HEK293 cells were treated with either miR-133a mimic (miR-133a) or scrambled miRNA (scm) with either WT CSE UTR or Mut CSE UTR, and relative luciferase activities were measured. We found that miR-133a decreased relative luciferase activity in WT CSE UTR with respect to scm, suggesting that miR-133a targets WT CSE UTR. Moreover, there was no change in luciferase activity after miR-133a mimic treatment to Mut CSE UTR (Fig. 3B) supporting that miR-133a targets CSE 3′UTR. To confirm that miR-133a target CSE, we performed RNA-EMSA (miRNA-mRNA interaction analysis by EMSA) using WT CSE UTR and Mut CSE UTR, a 22-mer RNA sequence for the 3′UTR corresponding to CSE, and miR-133a and anti-miR-133a probes. We performed the binding assays using the LNA miRNA-133a* sequence as the miRNA probe. In RNA-EMSA, miR-133a displays one band (Fig. 3C, lane 3). The incubation of miR-133a with an equimolar concentration of anti-miR-133a reduces the mobility of miR-133a band due to formation of miR-133a- anti-miR complex (Fig. 3C, lane 4). Similarly, 1 μM WT CSE UTR RNA displays single band (Fig. 3C, lane 5), however, incubation of different doses (2 μM and 4 μM) of WT CSE UTR RNA with miR-133a reduces electrophoretic mobility and form a second band of miR-133a-WT CSE UTR complex (Fig. 3C, lanes 6 and 7). This second band corresponds to the specific binding of the miR-133a with WT CSE UTR RNA (Fig. 3C, lane 6). Moreover, there was increased intensity of second band with increasing doses of CSE (Fig. 3C, lane 6 and 7, and 3D). The upper bands in lanes 6 and 7 represents the specific binding between miR-133a and WT CSE UTR RNA. To corroborate the specificity of miR-133a binding to CSE UTR, we also used 2 μM and 4 μM Mut CSE UTR, which had 7 mismatches with the target site of miR-133a. We observed no gel-shift in Mut CSE UTR suggesting an absence of miR-133a- Mut CSE UTR complex formation. Further, there was no second band for the two doses (2 μM and 4 μM) of Mut CSE UTR (Fig. 3C, lanes 8 and 9), demonstrating the specificity of miR-133a binding to CSE 3′UTR. Altogether, our results showed that miR-133a targets CSE.

### Dose-dependent effects of H_2_S and Hcy on miR-133a levels in cardiomyocytes

**Figure 4:**
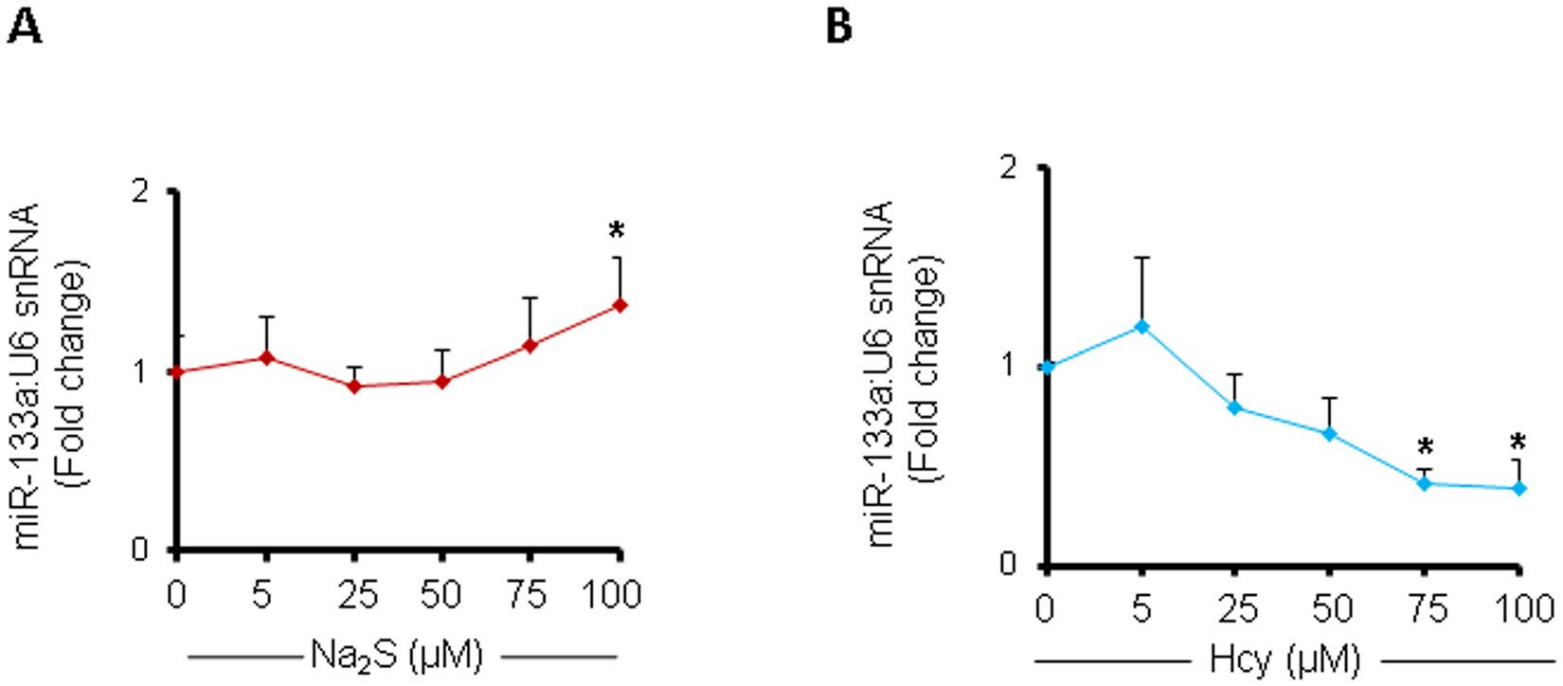
Dose-dependent effect of Na_2_S or Hcy on miR-133a levels in HL1 cardiomyocytes. (**A**) Individual miR-133a assay showing the dose-dependent effect of Na_2_S or (**B**) Hcy on miR-133a levels in cardiomyocytes. U6 is an endogenous control. Values are represented as mean ± SEM. “*” represents significant value when compared to the untreated control. p < 0.05 is considered statistically significant. N = 5.

To determine the dose-dependent effects of Na_2_S on miR-133a levels, we treated HL1 cardiomyocytes with the same doses of Na_2_S that was used for determining the CSE levels (Fig. 1D). We found no significant change in the levels of miR-133a when the cardiomyocytes were treated with the below 25 µM dose of Na_2_S. However, miR-133a level was markedly increased when treated with above 50 µM dose of Na_2_S (Fig. 4A). These results further support that Na_2_S have an indirect role, via upregulation of miR-133a, to suppress CSE in cardiomyocytes. Since Na_2_S increased miR-133a in a dose-dependent manner (Fig. 4A), we determined whether Hcy has a dose-dependent effect on miR-133a levels in cardiomyocytes. For that, we measured the levels of miR-133a after treatment with increasing doses of Hcy (Fig. 4B). Our results demonstrated that above 25 µM dose of Hcy, the levels of miR-133a was significantly decreased (Fig. 4B). Based on these findings, we infer that elevated levels of Hcy attenuates miR-133a in cardiomyocytes in a dose-dependent manner. Overall, our results suggest that Hcy and H_2_S have an opposite effect on miR-133a levels in cardiomyocytes.

### H_2_S mitigates homocysteine-mediated hypertrophy in cardiomyocytes

**Figure 5:**
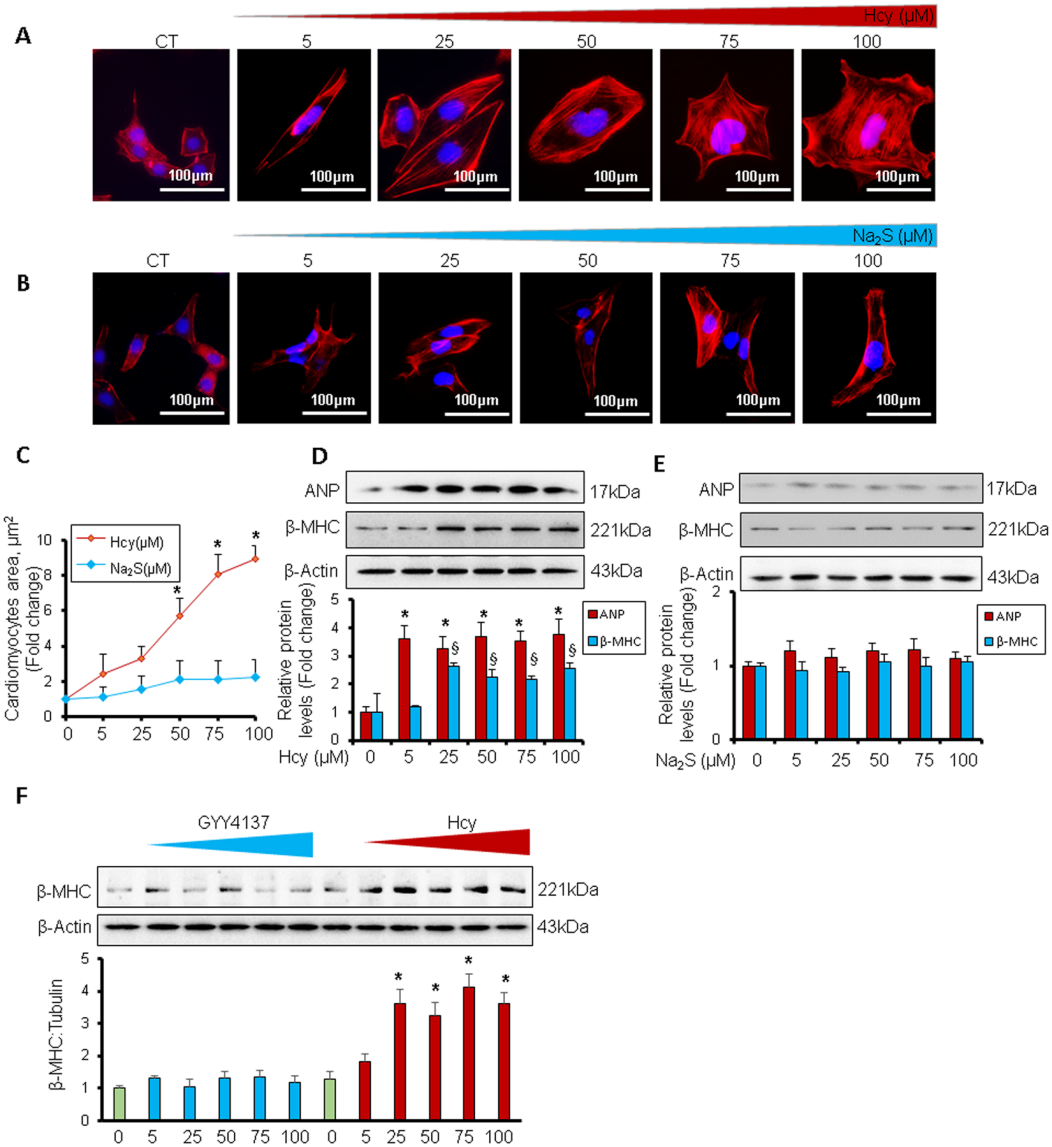
Dose-dependent effect of homocysteine or hydrogen sulfide donor Na_2_S/GYY4137 on hypertrophy in HL1 cardiomyocytes. (**A**) Representative F-actin staining for cardiomyocytes treated with different doses of Hcy, and (**B**) Na_2_S. (**C**) Quantification of cardiomyocytes area showing the increase in the area of cardiomyocyte surface after treatment with different dosages of Hcy or Na_2_S. Values are represented as mean ± SEM, n = 20 cells per group from three independent experiments. “*” Represents statistical significant value when compared to the untreated control. (**D**) Western blot analyses of atrial-natriuretic peptide (ANP) or beta-myosin heavy chain (β-MHC) after treatment with different dosages of Hcy or, (**E**) Na_2_S, or (**F**) GYY4137. Values are represented as mean ± SEM. “*” Represents statistically significant value for ANP when compared to the untreated control, and “§” represents statistically significant value for β-MHC when compared to the untreated control. p < 0.05 is considered statistically significant. Scale bar: 100 μm. N = 5.

**Figure 6:**
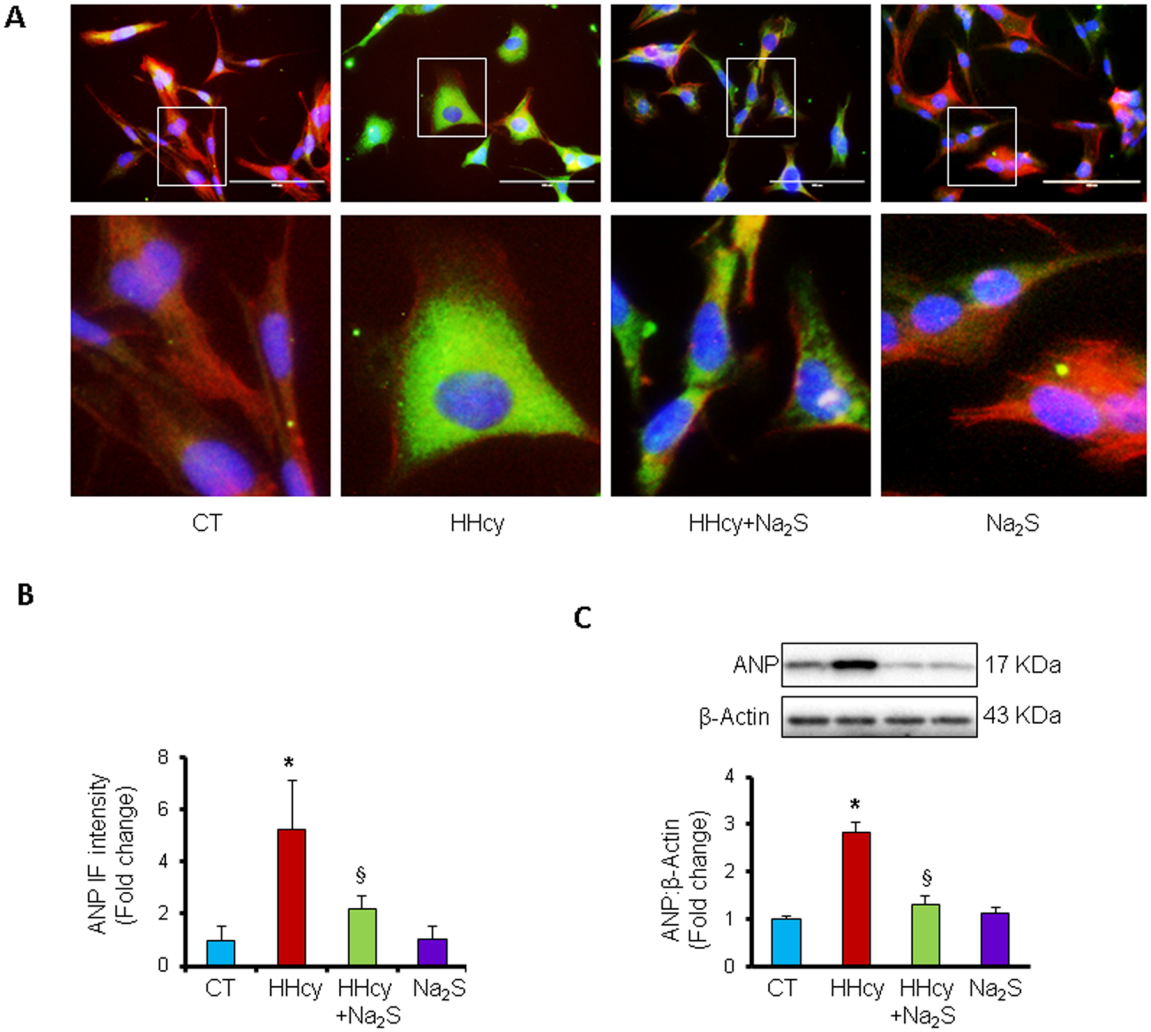
Hydrogen sulfide donor Na_2_S mitigates homocysteine-mediated upregulation of atrial-natriuretic peptide (ANP) in HL1 cardiomyocytes. (**A**) Representative immunocytochemistry of ANP (green) and β-tubulin (red) in cardiomyocytes treated with either nothing (CT), 100 µM of Hcy (HHcy), HHcy with 30 µM of Na_2_S (HHcy + Na_2_S), or 30 µM of Na_2_S (Na_2_S). n = 20 cells per group from three independent experiments. (**B**) Quantification of ANP immunofluorescence intensity in the above four groups of cardiomyocytes. (**C**) Western blot showing the relative expression of ANP. Values are represented as mean ± SEM. N = 5. p < 0.05 is considered statistically significant. “*” Represents statistically significant value when compared to the untreated control, and “§” represents statistically significant value when compared to the HHcy + Na_2_S treated cells. Scale bar: 100 μm.

**Figure 7:**
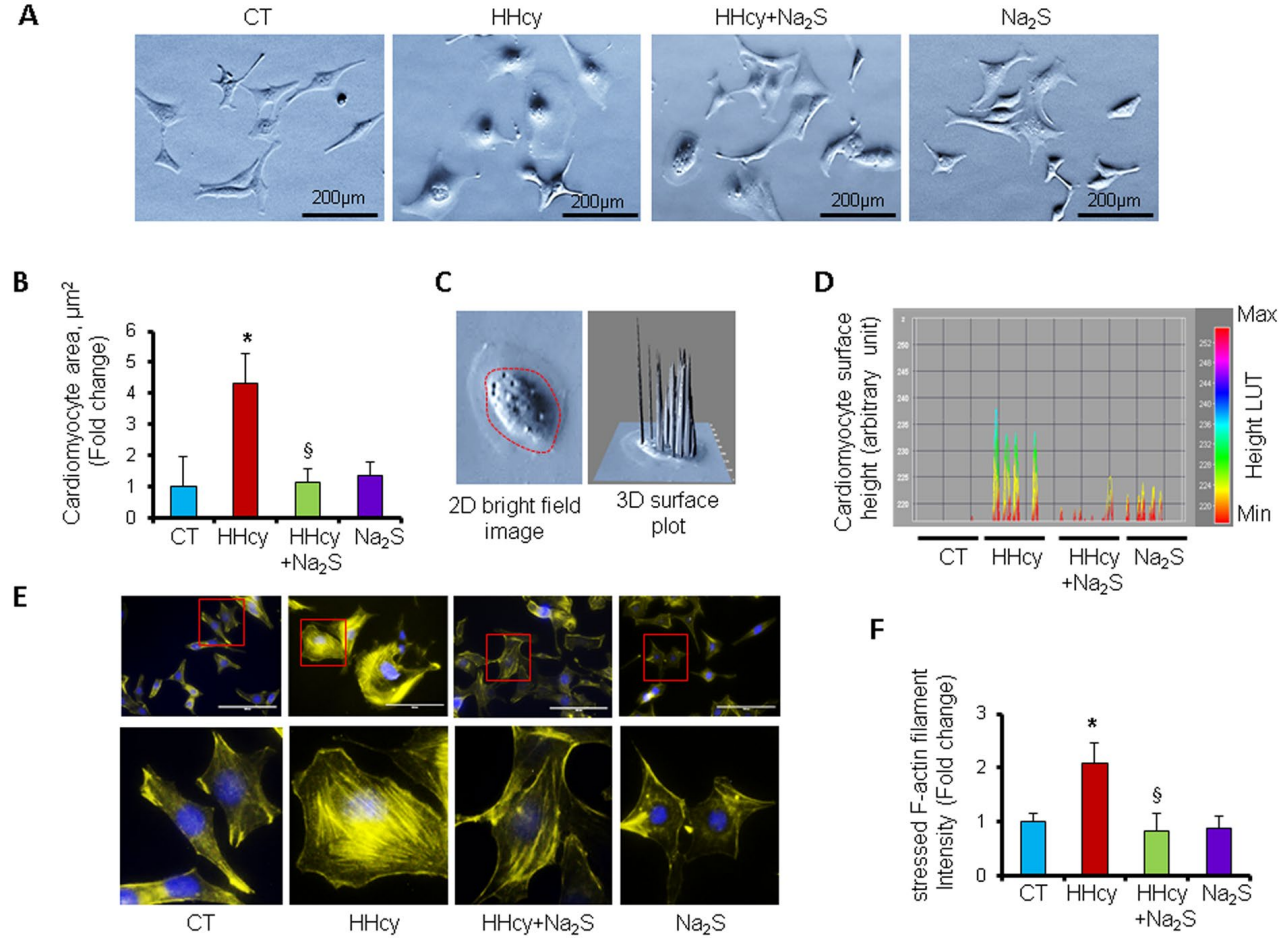
Hydrogen sulfide donor Na_2_S mitigates homocysteine-mediated increase in cardiomyocyte area and F-actin expression in HL1 cardiomyocytes. (**A**) Representative phase-contrast microscopic images showing the relative size of cardiomyocytes treated with either nothing (CT), 100 µM of Hcy (HHcy), HHcy with 30 µM of Na_2_S (HHcy + Na_2_S), or 30 µM of Na_2_S (Na_2_S). (**B**) Quantification of cardiomyocyte area in the above four groups of cardiomyocytes. Values are represented as mean ± SEM. n = 20 cells per group from three independent experiments. (**C**) Representative 2D phase-contrast and 3-dimentional (3D) shadow spike plot of a cardiomyocyte. (**D**) The shadow spikes converted lookup table (LUT) showing the arbitrary height of cardiomyocytes in the above four groups. (**E**) Representative F-actin staining on cardiomyocytes from the above four groups. (**F**) Quantification of intensity of F-actin from ‘E’. The increased F-actin intensity indicates cardiomyocyte hypertrophy. Values are represented as mean ± SEM, n = 20 cells per group. p < 0.05 is considered statistically significant. “*” Represents statistically significant values when compared to the untreated control, and “§” represents statistically significant values when compared to the HHcy + Na_2_S treated cells. Scale bars: A; 200 μm, E; 100 μm.

Since miR-133a is an anti-hypertrophic miRNA^18^, we sought to determine whether Na_2_S can attenuate Hcy-mediated hypertrophy in cardiomyocytes. For that, first we evaluated the dose-dependent effect of Hcy or Na_2_S on cardiomyocyte hypertrophy. We found that higher doses of Hcy induced cardiomyocyte hypertrophy but Na_2_S did not have effect on cardiomyocyte size (Fig. 5A–C). Moreover, we also confirmed cardiomyocyte hypertrophy by using molecular markers for hypertrophy such as atrial natriuretic peptide (ANP) and beta-myosin heavy chain (β-MHC). These molecular markers-based findings demonstrated that 25 µM or higher doses of Hcy caused hypertrophy in cardiomyocytes whereas Na_2_S did not change cardiomyocytes size (Fig. 5D,E). To corroborate the effect of H_2_S on hypertrophy, we treated HL1 cardiomyocytes with an alternate H_2_S donor GYY4137 using the same doses and measured the levels of beta-myosin heavy chain (β-MHC), a molecular marker for cardiomyocyte hypertrophy, to determine the cardiomyocyte hypertrophy. Our results showed that GYY4137 (same doses as Na_2_S, 5 µM–100 µM range) did not upregulates β-MHC, supporting that H_2_S donors do not induce cardiomyocyte hypertrophy. As a positive control, we also treated cardiomyocytes with increasing doses of Hcy, which showed upregulation of β-MHC at higher doses (above 25 µM) (Fig. 5F). These results provide a solid evidence that H_2_S have no effect on cardiomyocytes size in normal cardiomyocytes. However, it was unclear whether H_2_S will have same effect in HHcy cardiomyocytes. Therefore, we used the same molecular markers for hypertrophy and determined whether Na_2_S can mitigate Hcy-mediated cardiomyocyte hypertrophy. We treated HL1 cardiomyocytes with 100 µM of Hcy with or without 30 µM of Na_2_S, following our previously reported protocol^17^. Our results demonstrated that 100 µM of Hcy increased the protein (Fig. 6C) and cellular (Fig. 6A,B) levels of ANP but 30 µM of Na_2_S did not change the levels of ANP. Notably, Na_2_S blunted the upregulation of ANP in Hcy-treated cardiomyocytes (Fig. 6A–C). We further evaluated the effect of Hcy and/or Na_2_S on hypertrophy of cardiomyocytes by measuring the surface area of cardiomyocytes (Fig. 7A,B), using a 3D surface plot for cardiomyocytes (Fig. 7C,D), and by determining expression and intensity of F-actin in cardiomyocytes (Fig. 7E,F). We found that elevated levels of Hcy induces cardiomyocyte hypertrophy, which is mitigated by Na_2_S. Altogether, these results demonstrated that HHcy upregulates hypertrophy of cardiomyocytes and although H_2_S donor Na_2_S may not have an effect on hypertrophy in normal cardiomyocytes, it attenuates Hcy-mediated hypertrophy of cardiomyocytes.

### CBS deficiency upregulates CSE by inducing SP1 in the mouse heart

**Figure 8:**
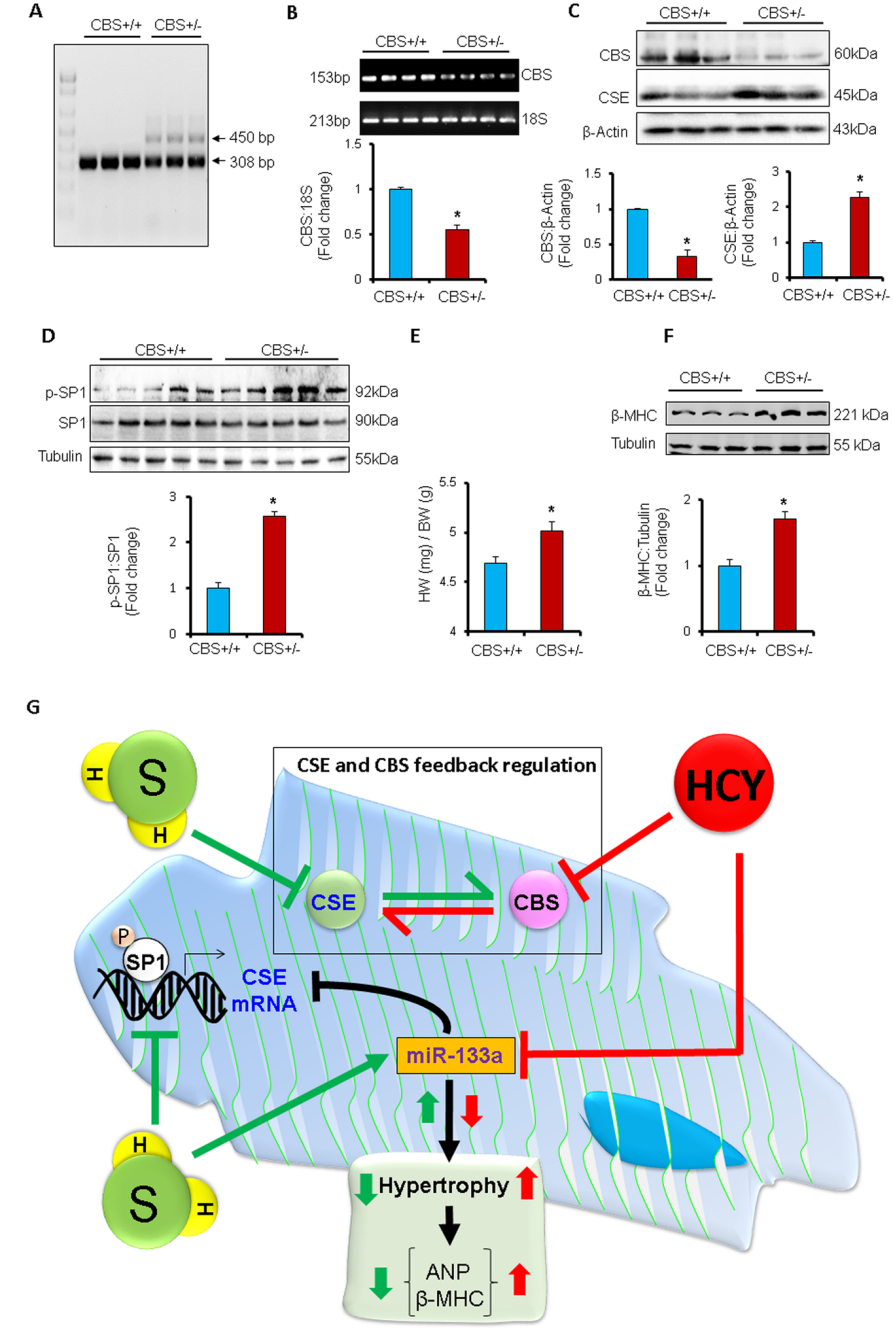
Deficiency of CBS upregulates CSE by inducing SP1. (**A**) Genotyping for CBS+/− and CBS+/+ mice. Representative agarose gel electrophoresis showing PCR bands of CBS+/− and CBS+/+ mice. CBS heterozygous (CBS+/−) mice have two bands whereas CBS homozygous (CBS+/+) mice have single band. N = 3. (**B**) RT-PCR showing cardiac levels of CBS in CBS+/− and CBS+/+ mice. 18 S is an endogenous control. N = 4. (**C**) Western blot showing cardiac levels of CBS and CSE in CBS+/+ and CBS+/− mice. N = 5. (**D**) Western blot showing the activity of SP1 (p-SP1: SP1) in the heart of CBS+/+ and CBS+/− mice. N = 5. Values are represented as mean ± SEM. p < 0.05 is considered statistically significant. (**E**) Bar graph showing heart to body weight ratio in CBS+/+ and CBS+/− mice ( p = 0.01). (**F**) Western blot showing the cardiac expression of β-myosin heavy chain (β-MHC), a molecular marker for cardiac hypertrophy, in CBS+/+, and CBS+/− mice. N = 5. Values are represented as mean ± SEM. p < 0.05 is considered statistically significant. “*” Represents statistically significant values when compared to the CBS+/+ control. (**G**) Schematic representation showing the negative feedback regulation of CBS and CSE, and impact of homocysteine (Hcy) or hydrogen sulfide (H_2_S) on this feedback regulation. Hcy downregulates CBS and miR-133a. Reduced levels of miR-133a induces cardiac hypertrophy, which is reflected by increased levels of atrial natriuretic peptide (ANP) and beta-myosin heavy chain (β-MHC), the molecular markers for cardiac hypertrophy. H_2_S inhibits hypertrophy by inducing miR-133a. MiR-133a also targets SP1, an inducer of CSE. Therefore, H_2_S indirectly downregulates SP1 by upregulating miR-133a. H_2_S also inhibits CSE directly by suppressing the activity of SP1.

*In vitro* studies showed that Hcy downregulates CBS but upregulates CSE (Fig. 1A,B) whereas H_2_S donor Na_2_S upregulates CBS but downregulates CSE (Fig. 1C,D), indicating that when CBS is upregulated CSE is downregulated or vice versa. To determine whether CBS has a direct effect on CSE, we used CBS deficient (CBS+/−) and a sibling control (CBS+/+) mice. First, we validated these mice by genotyping (Fig. 8A), and mRNA expression in the heart (Fig. 8B). Then we measured the protein levels of CBS and CSE in the heart. We found that cardiac levels of CSE was upregulated but CBS was downregulated in CBS+/− as compared to CBS+/+ mice (Fig. 8C). These results showed that CBS deficiency induces CSE in the heart. We also measured the activity of SP1, which upregulates CSE^12, 13^, and found that SP1 activity (ratio of p-SP1: SP1) was increased in CBS+/− hearts (Fig. 8D). It suggests that CBS deficiency upregulates CSE plausibly by inducing SP1 activity in the CBS+/− hearts. We also determined cardiac hypertrophy in these mice by measuring heart to body weight ratio (Fig. 8E) and determining β-MHC levels in the heart (Fig. 8F). Our results support that CBS deficiency induces cardiac hypertrophy in mice (Fig. 8E,F). Overall, these findings revealed a negative feedback regulation of CBS and CSE in cardiomyocytes/hearts, which can be influenced by HHcy or H_2_S donor Na_2_S/GYY4137.

## Discussion

Although the physiological effect of H_2_S on the heart^20–22^, and the underlying molecular mechanisms are documented^10, 23–25^, the differential effect of H_2_S or Hcy on CBS and CSE expressions, the cross-talk/feedback regulation of CBS and CSE, and the underlying molecular mechanism by which H_2_S regulates CSE are unclear. The present study fill-in the gap of knowledge in the field of H_2_S and Hcy biology by answering these questions. We reveal a novel negative feedback regulation of CBS and CSE, where deficiency of CBS upregulates CSE, plausibly by inducing SP1. The elevated levels of Hcy or H_2_S donor Na_2_S/GYY4137 influence this regulation. HHcy suppresses CBS that results in upregulated CSE. HHcy also downregulates anti-hypertrophic miR-133a causing cardiomyocyte hypertrophy. On contrary, H_2_S suppresses CSE directly by inhibiting SP1 and indirectly by inducing miR-133a that targets CSE, which results in CBS upregulation. H_2_S also mitigates Hcy-mediated hypertrophy of cardiomyocytes by increasing the levels of miR-133a (Fig. 8G).

Hcy is biosynthesized from methionine, an essential amino acid, by two enzymes, s-adenosyl methionine and s-adenosyl homocysteine that transfer methyl groups from methionine to convert it into Hcy. Hcy remethylates to methionine by methyl tetrahydrofolate enzyme and folic acid cofactor, or metabolizes to cysteine and H_2_S by CBS and CSE enzymes through transsulfuration pathway^8^. If remethylation or transsulfuration pathway is impaired, Hcy is accumulated and circulating levels of Hcy is elevated (HHcy). As per The American Heart Association advisory statement, the normal homocysteine level in the blood ranges from 5–15 µmol L^−1^, however, more than 9 µmol L^−1^ is associated with mortality of patients^3^. The increasing doses of Hcy can be classified into mild HHcy, where Hcy level in the blood ranges from 9–14.9^3, 26, 27^, intermediate HHcy which ranges from 31–100 µmol L^−1^, and severe HHcy where Hcy levels are more than 100 µmol L^−1^ and it is associated with inborn error in Hcy metabolism^28^. Previous studies on Hcy-mediated cardiomyocyte hypertrophy focused on higher dose of Hcy^17, 29, 30^. However, the dose-dependent effect of Hcy on cardiomyocyte hypertrophy was unclear. In the present study, we used different doses of Hcy and demonstrate that even 5 µmol L^−1^ of Hcy can increase size of cardiomyocytes, and there is a linear relationship with increasing doses of Hcy with cardiomyocyte hypertrophy when the Hcy dose is higher than 25 µmol L^−1^ (Fig. 5C). Since Hcy is transsulfurated into H_2_S^8^, and H_2_S induces anti-hypertrophy miR-133a^17^, we also measured the effect of H_2_S on cardiomyocyte hypertrophy. Interestingly, increasing doses of H_2_S donor Na_2_S did not have an effect on cardiomyocyte size and there was no hypertrophy in Na_2_S-treated cardiomyocytes (Fig. 5C and E). It suggests that H_2_S may not influence hypertrophic pathway in normal cardiomyocytes. However, in Hcy-treated cardiomyocytes, we observed that even low levels of H_2_S mitigates hypertrophy of cardiomyocytes (Figs 6 and 7) implying that H_2_S acts as anti-hypertrophy when cardiomyocyte hypertrophy is instigated by pathological insult such as HHcy. These findings are consistent with previous report demonstrating that increased level of Hcy causes cardiomyocyte hypertrophy^17, 29^, H_2_S blunts Hcy-mediated cardiomyocyte hypertrophy^17^, and cardiac hypertrophy is suppressed by miR-133a^18, 31^.

Hcy is involved in pathological cardiac remodeling^32–35^, and miR-133a prevents pathological remodeling by suppressing cardiac hypertrophy^18, 31^ and fibrosis^16, 36^. Although H_2_S upregulates miR-133a in cardiomyocytes^17^, the dose-dependent effect of Hcy or H_2_S on miR-133a is unclear. In the present study, we demonstrate that above 5 µM of Hcy, the increasing doses of Hcy is associated with decreasing levels of miR-133a in cardiomyocytes (Fig. 4B). On contrary, above 50 µM of Na_2_S, increasing doses of Na_2_S is associated with increasing levels of miR-133a in cardiomyocytes (Fig. 4A). These results elicit the dose-dependent effect of Hcy or H_2_S on miR-133a levels in cardiomyocytes, which may be important for future studies on Hcy or H_2_S-mediated cardiac remodeling. We have demonstrated that elevated levels of H_2_S increases miR-133a (Fig. 4A), whereas HHcy reduces miR-133a (Fig. 4B). Since miR-133a is anti-hypertrophic to the cardiomyocytes/heart^19, 37, 38^, we proposed that H_2_S upregulates miR-133a, whereas Hcy downregulates miR-133a, and differentially regulate cardiomyocytes hypertrophy (Fig. 8G). The effect of H_2_S on upregulation of miR-133a is not novel and is previously reported by our group^17^ and others^39^. The present results support the fact that H_2_S-mediated upregulation of miR-133a reduces cardiomyocyte hypertrophy (Figs 5A–7F). Moreover, our group has shed light on the underlying molecular mechanism by which H_2_S mitigates Hcy-mediated downregulation of miR-133a in cardiomyocytes^17^. In the present study, we also observed that H_2_S blunts the effect of Hcy on cardiomyocytes hypertrophy (Figs 5A–7F).

Hcy is converted into H_2_S by CBS and CSE enzymes. CBS converts Hcy into cystathionine, which is then converted into cysteine by CSE that ultimately biosynthesize H_2_S^8^. However, whether Hcy or H_2_S has a dose-dependent effect on either CBS or CSE is unclear. Our results demonstrate that above 25 µM, the increasing doses of Hcy upregulates CSE and downregulates CBS (Fig. 1A,B). It suggests that HHcy suppresses CBS but induces CSE. On contrary, higher (25–75 µML^−1^) doses of H_2_S downregulates CSE but upregulates CBS (Fig. 1C,D). To our knowledge, this is the first report showing the yin-yang effect of Hcy versus H_2_S on CBS and CSE levels in cardiomyocytes.

H_2_S is a cardioprotective gaseous molecule^10, 21, 40–44^, and is emerging as a novel therapeutic target for heart failure^45^ (clinicaltrials.gov; No. NCT02180074). A wide range (15–300 µM) of physiological levels of H_2_S levels has been reported *in vivo*, perhaps due to variable detection methods^10^. In the present study, we used a range of 0–100 µML^−1^, which is within the *in vivo* levels of H_2_S documented in the literature^10^. H_2_S is also emerged as a signaling molecule^24^, and biosynthesis of H_2_S from CBS and CSE enzymes is established^8, 10, 24^. However, whether H_2_S has any effect on CBS or CSE is poorly understood. In the present study, we reveal that lower levels (0–25 µM) or high level (100 µML^−1^) of Na_2_S may not have a significant effect on CBS or CSE levels in normal cardiomyocytes (Fig. 1C,D). The effective dose range for Na_2_S to have an impact on CBS or CSE expression in normal cardiomyocytes is between >25 µML^−1^ and <100 µML^−1^ (Fig. 1C,D). Although these results are encouraging, this concept needs further evaluation and validated at *in vivo* level.

H_2_S has an inhibitory effect on CSE at higher doses (Fig. 1C–E), however, the underlying molecular mechanism is unclear. Here, we demonstrate that H_2_S downregulates CSE by directly suppressing SP1 activity (Fig. 2A), which is an inducer of CSE^12–14^. On contrary, Hcy induces SP1 activity at higher doses by increasing its binding to CSE promoter (Fig. 2B–D). It is documented that exogenous treatment with H_2_S donor Na_2_S upregulates miRNAs^46, 47^, a non-coding regulatory RNA that has emerged as a therapeutic target for cardiovascular diseases^48^. Na_2_S treatment upregulates miR-21 that targets CSE^46^. The other miRNAs involved in regulation of CSE are miR-30 that directly inhibits CSE^47^, and miR-22 that inhibits SP1^49^. However, effect of miR-133a, the most abundant miRNA in the heart^50^, on CSE was unknown. In the present study, we reveal that miR-133a targets CSE (Fig. 3A–D). Notably, H_2_S may indirectly reduce CSE by upregulating miR-133a (Fig. 4A). Hcy has an opposite effect on miR-133a levels and increasing doses of Hcy downregulates miR-133a (Fig. 4B). Altogether, we show a novel mechanism for H_2_S-mediated regulation of CSE in cardiomyocytes, where it can directly inhibit CSE by suppressing SP1 and indirectly reduce CSE levels by upregulating miR-133a that targets CSE.

Although Hcy and H_2_S have opposite effects on CBS and CSE expression in cardiomyocytes (Fig. 1A–E), it was unclear whether CBS directly influences CSE expressions in the heart. Our results demonstrate that deficiency of CBS upregulates cardiac CSE in CBS+/− mice (Fig. 8A–C). Moreover, we reveal the underlying molecular mechanism for CBS-mediated regulation of CSE in the heart. Deficiency of CBS induces SP1 activity (Fig. 8D), an inducer for CSE that results in elevated levels of CSE in the heart (Fig. 8C). It is a novel mechanism for CBS-mediated regulation of CSE. Based on these findings, we propose a novel negative feedback regulation between CBS and CSE in the heart (Figs 1A–E and 8C). CBS deficient mice cannot transsulfurate Hcy into H_2_S that elevates Hcy levels. Lack of CBS induces cardiac hypertrophy (Fig. 8E,F); plausibly via HHcy, that reduces anti-hypertrophic miR-133a (Fig. 4B).

Cardiac hypertrophy is associated with pathological cardiac remodeling and involves several signaling pathways^51^. MiR-133a mitigates cardiac hypertrophy by targeting RhoA, a cardiac hypertrophy regulating protein, and cdc42, a kinase involved in hypertrophy^18^. We have reported that Hcy-treatment reduces the levels of miR-133a in cardiomyocytes and induces c-fos, an early marker for hypertrophy, in HL1 cardiomyocytes^17^. Hcy also regulates ERK pathway^30^ and ATP7a^29^ to control cardiac hypertrophy. In the present study, we demonstrate that Hcy-induced cardiomyocyte hypertrophy, reflected by the morphology of cardiomyocytes and molecular markers of hypertrophy, such as ANP and β-MHC, can be normalized by Na_2_S treatment (Figs 5A–7F).

H_2_S is a volatile gaseous molecule with a short half-life^10^ whereas Hcy is a stable amino acid. Hence, there is a possibility that H_2_S levels are not maintained in the culture medium for 24-hour treatment period but Hcy levels maintained for 24-hour treatment period. Therefore, in the experimental group where HL1 cardiomyocytes were treated with both Na_2_S and Hcy, the effect of Na_2_S may be ephemeral but that of Hcy is prolonged. It is documented that even 30 minutes of H_2_S donor pre-treatment is able to mitigate phenylepinephrine-mediated hypertrophy in cardiomyocytes by upregulating anti-hypertrophic miR-133a^39^. MiR-133a transcription is regulated by myocyte enhancer factor-2C (Mef2c)^52^. We have reported that Hcy inactivates Mef2c by promoting the binding of Mef2c with HDAC1 whereas Na_2_S activates Mef2c by releasing Mef2c from HDAC1 in HL1 cardiomyocytes^17^. For this reason even the short-term presence of H_2_S may be adequate to induce miR-133a transcription in Hcy-treated cardiomyocytes, and high levels of miR-133a is able to inhibit cardiomyocyte hypertrophy even in the absence of H_2_S.

Overall, we demonstrate a novel yin-yang effect of H_2_S versus Hcy on CBS and CSE in the heart using *in vitro* and *in vivo* approaches. It was interesting to note that dose-dependent effect of Hcy and H_2_S are unique for CBS, CSE, and miR-133a, and the dose effect of H_2_S in normal cardiomyocytes and Hcy-treated cardiomyocytes are different. We also revealed a novel molecular mechanism for H_2_S-mediated inhibition of CSE in cardiomyocytes, where H_2_S directly inhibits SP1, an inducer for CSE, to suppress CSE and indirectly upregulates miR-133a that targets CSE. The upregulation of miR-133a by H_2_S also mitigates Hcy-mediated hypertrophy of cardiomyocytes. Our results from CBS+/− and CBS+/+ hearts demonstrate a negative feedback regulation mechanism between CBS and CSE in the heart. These findings are important for understanding the underlying molecular mechanism of Hcy-, or H_2_S-mediated cardiac remodeling, including hypertrophy, which could be crucial for developing a H_2_S-based therapeutic strategy.

### Limitations

Our experiments are performed on HL1 cardiomyocytes cell line, CBS+/− and CBS+/+ mice. These results need further validation at *in vivo* levels by treating CSE+/− mice with a H_2_S donor such as Na_2_S or GYY4137, and treating pathological hearts such as pressure-overload or volume overload hearts with a H_2_S donor. Since the effect of H_2_S concentration on cellular activity is poorly understood^24^ and a high dose of H_2_S could be toxic to the cell, treatment with a slow H_2_S-releasing donor such as SG1002 can be a better approach for *in vivo* experiments to assess the effect of H_2_S on CBS, CSE, miR-133a, and cardiac remodeling in the pathological hearts. It is unclear that how much Hcy or H_2_S enters into the cardiomyocytes after treatment with Hcy or Na_2_S. Examining the levels of intracellular Hcy or H_2_S and correlating that with the expression of CSE, CBS, and miR-133a will provide more clarity to the cross-talk among these molecules. Even though we have elucidated the underlying mechanism for H_2_S-, and Hcy-mediated regulation of CBS, CSE, and miR-133a, further molecular studies are required to uncover the regulatory signaling mechanisms.

## Materials and Methods

We procured CBS+/− male and female mice from the Jackson Laboratory (Bar Harbor, ME USA, Stock # 002853) and bred these mice in the animal facility of the University of Nebraska Medical Center to obtain CBS+/+ mice. Mice were kept in the animal facility in a room with temperature 22–24 °C, humidity 30–40% humidity, and 12 hours’ light/dark cycle, and food and water were supplied to them ad libitum. For experiments, twelve-week male mice were used. All the experimental protocols on mice were approved by the Institutional Animal Care and Use Committee of the University of Nebraska Medical Center, and all methods were performed in accordance with the relevant guidelines and regulations.

### Genotyping of CBS+/− mice

Genomic DNA was extracted from the ear punch tissue of mice at the age of four to six week and was amplified by polymerase chain reaction (PCR) using the protocol provided by the Jackson Laboratory. The forward primer sequence which is common for CBS+/+ and CBS+/− was 5′GATTGCTTGCCTCCCTACTG3′. The reverse primer sequence for CBS+/+ was 5′AGCCAACTTAGCCCTTACCC3′, and for CBS+/− was 5′CGTGCAATCCATCTTGTTCA3′, respectively. PCR product for CBS+/+ mice had one band at 308 bp whereas PCR product for CBS+/− had two bands at 308 bp and 450 bp (Fig. 8A).

### *In vitro* studies

We used mouse atrial HL1 cardiomyocytes and cultured them following the established protocol^53^. These cardiomyocytes were cultured in a 6-well plate. At 70–80% confluency cells were treated with Hcy (cat # H4628, Sigma, USA) with or without Na_2_S (cat # 407410, Sigma, USA) for 24 hours. The treatment groups were: no treatment control (CT), 100 μmL^−1^ Hcy (HHcy), HHcy and 30 μmL^−1^ of Na_2_S (HHcy + Na_2_S), and 30 μmL^−1^ of Na_2_S. For assessing the dose-dependent effect of Hcy or Na_2_S, or a slow-releasing H_2_S donor, we treated HL1 cardiomyocytes separately with 0, 5, 25, 50, 75, or 100 μmL^−1^ of either Hcy or Na_2_S, or GYY4137 (cat # SML0100, Sigma, USA) respectively.

### RNA isolation and miRNA-133a assay

Total RNA was isolated using mirVana™ miRNA Isolation kit (cat # AM1560, Life technologies, USA)^34^. The purity and quantity of RNA was measured by NanoDrop 2000c (Thermo Scientific Inc., USA), and good quality RNA was used for miR-133a assay as described elsewhere^19^.

### Reverse Transcription Quantitative PCR (RT-QPCR)

For quantitative amplification of mRNA, we used 1 μg of total RNA and iScript™ Reverse Transcription Supermix (cat # 1708841, Bio-Rad Laboratories, USA) to synthesize cDNA, which was then amplified using SYBR green in a C1000 Touch thermal cycler (Bio-Rad Laboratories, USA) following manufacturer’s instructions^19^. We used 18sRNA as an endogenous control. The primer sequences were: ANP (NM_008725), forward 5′CTGCCTCATTAATGCTTAC3′, reverse 5′TGGCTGTTATCTTCGGTAC3′, 18sRNA (NR_003278), forward 5′GTAGTTCCGACCATAAACGA3′ and reverse 5′TCAATCTGTCAATCCTGTCC3′, CSE (AY262829), forward 5′TGCCTCACCCCATTTCATCT3′ and reverse 5′GAGTAAACTGGGTGAGGGCT3′, and CBS (NM_144855) forward 5′TGCGGAACTACATGTCCAAG3′ and reverse- 5′TTGCAGACTTCGTCTGATGG3′. The melting temperature for all reactions was 55 °C. For qualitative PCR of CSE, cDNA was amplified in a BioRad cycler, and electrophoresis was performed in a 2% agarose gel. The gel was developed by a Chemidoc (Bio-Rad Laboratories, USA), and band intensity was quantified by a ChemiDoc software Image Lab 4.1 (Bio-Rad Laboratories, USA).

### Western Blotting

Standard protocol for Western blotting was used^19^. Protein was extracted from HL1 cardiomyocytes and heart tissue by using radio-immuno-precipitation assay lysis buffer (RIPA, cat # BP-115D, Boston BioProducts, USA), and quantified by using BCA protein assay kit (cat # 23227, Pierce, USA). Equal amounts (30 µg) of proteins were used for SDS-PAGE. The primary antibodies used were: β-MHC (cat # ab172967, Abcam, USA), ANP (cat # GTX 109255, Gentex, USA), CBS (cat # H00000875-M01, Abnova, USA), CTH (cat # H00001491-M02, Abnova, USA), β-actin (cat # sc-47778, Santa Cruz Biotechnology, USA), β-tubulin (cat # MA5-16308, Thermo Scientific Inc., USA), p-SP1 (cat # ab59257, Abcam, USA), and SP1 (cat # 07-645, Millipore, USA). The secondary antibodies used were: anti-mouse IgG-HRP, and anti-rabbit IgG-HRP (cat # sc-2005, and cat # sc-2054, respectively, Santa Cruz Biotechnology, USA). Restore™ PLUS Western Blot stripping buffer (cat # 46430, Thermo Scientific Inc., USA) was used for restriping and reprobing of blots. Western blot membrane was developed by a Clarity™ Western ECL Substrate (cat # 1705061, Bio-Rad Laboratories, USA), imaged by a Chemidoc (Bio-Rad Laboratories, USA), and band intensity was analyzed by a ChemiDoc software Image Lab 4.1 (Bio-Rad Laboratories, USA).

### Immunocytochemistry

Standard protocol was followed for immunocytochemistry of HL1 cardiomyocytes^17^. In brief, medium from 60% confluent HL1 cardiomyocytes was removed and quickly washed with PBS, and fixed with 4% (w/v) paraformaldehyde pH 7.4 (cat # 158127, Sigma, USA) for 40 minutes at room temperature. They were then washed in PBS, permeabilized with 0.01% (v/v) Triton-X-100 for 30 minutes, and blocked with 1% (w/v) BSA solution (cat # A7030, Sigma, USA) for one hour. They were then incubated with primary antibody (1: 200 dilution) overnight at 4 °C. The primary antibodies used for immunocytochemistry were: ANP (cat # AB5490, Millipore, USA), and β-tubulin (cat # sc-5274, Santa Cruz Biotechnology, USA). Next day, they were washed with 0.02% PBST (PBS in Tween-20), and incubated with fluorescence conjugated secondary antibody (1:400 dilution) for one hour in dark. The secondary antibodies were: anti-rabbit AlexaFluor-488 and anti-mouse AlexaFluor-594 (cat # A21441 and A21201, respectively, Life Technologies, USA). After washing again with PBST, they were counterstained with DAPI (cat # A1001, AppliChem, USA, diluted 1:100 from a 1 µg/ml DAPI stock) for one minute. They were then washed with PBST, and mounted with Fluoromount mounting medium (cat # F4680, Sigma, USA), and observed under EVOS Cell Imaging Systems (Life technologies, USA). The intensity of color was analyzed by Image J software (NIH, USA).

### F-actin staining

Similar to immunocytochemistry, cardiomyocytes were fixed with 4% (w/v) paraformaldehyde, pH 7.4 (cat #158127, Sigma, USA) for 40 minutes at room temperature, and permeabilized with 0.5% (v/v) Triton-X-100 in PBS for 30 minutes. They were then incubated with 200 μM of Alexa Fluor® 594 Phalloidin (cat # A12381, Life Technologies, USA) in PBS for 20 minutes in the dark, and washed with PBST. They were observed under EVOS Cell Imaging Systems (Life technologies, USA). The intensity of F-actin color was analyzed by Image J software (NIH, USA).

### Luciferase reporter assay

We performed luciferase reporter assay on HEK293 cells. These cells were transfected with either GFP-tagged miR-133a (cat # MmiR3445-MR03) or GFP-tagged scrambled miRNA (cat# CmiR0001-MR03), which were purchased from GeneCopoeia, Rockville, MD, USA. We received custom designed CSE 3′ untranslated region (UTR) clones (WT 3′UTR: cat # MmiT038666-MT06; mutant 3′UTR: CS-MmiT038666-MT06-01) from GeneCopoeia, Rockville, MD, USA. For luciferase reporter assay we treated 1 μg of WT or mutant CSE to either GFP miR-133a or GFP-scrambled miRNA expressing cells for 48 hours, and measured the relative luciferase activity using Dual-Glow Luciferase Assay kit (cat # E2920, Promega Corp., Madison, WI) and a GloMax-Multi + Detection System (Promega) following the manufacturer’s manual.

### Measurement of cardiomyocyte surface area

The surface area of a cardiomyocyte was measured using the Image J (NIH) software that generates a 3-dimentional (3D) surface plot on a phase contrast image of a cardiomyocyte. In 3D image, the shadow spikes were transformed into lookup table (LUT) which correspond to cardiomyocytes arbitrary height. Moreover, individual cardiomyocyte surface area was also measured by using Alexa Fluor® 595 Phalloidin staining and the color intensity was quantified by the Image J software.

### Electrophoretic mobility shift assay (EMSA)

We treated HL1 cells separately with 0, 5, 25, 50, 75, or 100 μmL-1 of Hcy for 24 h in incomplete Claycomb medium. After that, cells were washed with ice-cold phosphate-buffered saline (PBS) and harvested using trypsin. They were pelleted in 1 ml ice-cold PBS by centrifugation at 1000 × g for 5 min at 4 °C, suspended in 500 μl of lysis buffer (50 mM KCl, 0.5% NP40, 25 mM HEPES PH 7.8, and 125 μm DTT supplemented with protease inhibitor cocktail; Sigma, USA), and kept for 15 min at 4 °C to rupture the cell membrane. The ruptured cells were then centrifuged at 10000 × g for 1 min at 4 °C to separate nuclei from the cytoplasmic fraction. The supernatant was removed and the pellet was suspended in 500 μl of washing buffer (50 mM KCl, 25 mM HEPES PH 7.8, 125 μm DTT supplemented with protease inhibitor cocktail), and centrifuged at 10000 × g for 1 min at 4 °C to collect the pellet that contains nuclear fraction. The nuclear pellet was suspended in 100 μl of nuclear extraction buffer (500 mM KCl, 25 mM HEPES pH 7.8, 50% glycerol and 125 μm DTT supplemented with protease inhibitor cocktail) and incubated at 4 °C for 15 min with constant rocking. The nuclear lysate obtained by this process was centrifuged at 1000 × g at 4 °C and the supernatant was collected as nuclear extract. We estimated the protein concentration of nuclear extract by BCA protein assay kit (Thermo Scientific Inc., USA) and nuclear protein was immediately processed for EMSA. We used 5 μg of nuclear protein and incubated it with mouse CSE promoter-specific SP1 response elements (WT 5′GAGGCGGGGC3′ and mutant 5′GATTCGGGGC3′ as per the previous report^54^ for 30 min at room temperature. The EMSA probes used were; WT 5′GCCACTGGGAGGCGGGGCAGGAACGATC3′ and Mutant 5′GCCACTGGGATTCGGGGCAGGAACGATC3′ and its complementary oligonucleotides (IDT, USA). We used fluorescence-based EMSA Kit (cat # E33075, Thermo Scientific Inc., USA) and labelled the oligonucleotides with SYBR green EMSA nucleic acid gel stain. For CSE-SP1 complex retardation, we used p-SP1 antibody (cat # ab59257, Abcam, USA). Reaction mixtures were loaded on a 6% polyacrylamide gel and gel electrophoresis was performed in 0.5X TBE buffer (pH 8) at 200 V for 1 h. The SYBR stained gel was scanned in a Chemidoc (ChemiDoc, Image Lab 4.1, Bio-Rad Laboratories, USA), using SYBR green filter with UV trans-illumination.

### Chromatin Immunoprecipitation (ChIP) assay

We treated HL1 cells separately with 0, 5, 25, 50, 75, or 100 μmL^−1^ of Hcy for 24 h in incomplete Claycomb medium. Cells were harvested using trypsin and then washed twice with ice-cold PBS followed by centrifugation at 1000 × g for 5 min at 4 °C. Cells were cross-linked by adding formaldehyde to a final concentration of 1% (v/v) to 1 ml cell suspension and incubated for 7 min at room temperature with gentle rotation. The cross-linked cells were prepared for ChIP assay using Zymo-Spin ChIP kit (cat # D5209, Zymo Research, USA) following the manufacturer’s instructions. In brief, cells were resuspended in nuclear pellet preparation buffer containing protease inhibitors. Nuclear pellets were sonicated on ice to shear chromatin into 200 to 300 bp in size using a sonication (Bioruptor, USA) in chromatin shearing buffer. The sheared chromatin supernatant was diluted with chromatin dilution buffer and incubated with p-SP1 (cat # ab59257, Abcam, USA), or normal rabbit IgG polyclonal antibody for overnight at 4 °C with constant rotation. An aliquot of the sheared chromatin was set aside for use as an input control. The chromatin-antibody complexes are precipitated with Zymomag Protein A beads, and chromatins were eluted, reversed cross-linked, and processed for ChIP DNA purification. The target CSE promoter fragment containing SP1 binding sites was amplified by qPCR using mouse CSE-specific oligonucleotide primers. The primer sequences used were: forward 5′CGGTACCTCTGTGCCACTGGGAG3′ and reverse 5′GAAGCTTGAGTGCGAGGTGTTGCT3′ as per published report^55^. The amplification of CSE promoter copy number was quantified by qPCR and was normalized by using control cells without Hcy treatment. The input was used as a positive control for the ChIP assay.

### MiRNA-mRNA Electrophoretic mobility shift assay (miRNA-mRNA EMSA)

We followed the published protocol for the miRNA-mRNA EMSA^56^. For binding assay Locked Nucleic Acid (LNA) miRNA-133a*, and RNA oligonucleotides were custom synthesized (Exiqon, USA). The sequences used were LNA mmu-miR-133a* 5′GCTGGTAAAATGGAACCAAAT3′, mmu-CSE WT UTR; 5′rGrArArArArArUrUrArUrArUrArArUrUrArCrCrArUrA3′, and mmu-CSE mutant UTR 5′rGrArArArArArArArArUrUrCrArArUrArUrCrUrArUrA3′. LNA-mmu-miR-133a* were incubated in EMSA binding buffer (10 mM MgCl2, 100 mM NaCl, 50 mM HEPES pH 7.2 and 5% glycerol) for 30 min at 37 °C with corresponding WT or mutant CSE RNA oligonucleotides. Binding reactions were electrophoresed in a 12% PAGE (10 mM MgCl2, 50 mM HEPES pH 7.2 and 5% glycerol) for 2 h at 190 V at 4 °C. EMSA was performed using fluorescence-based EMSA Kit (cat # E33075, Thermo Scientific Inc., USA) and labeling of the oligonucleotides was performed with SYBR green EMSA nucleic acid gel stain. The SYBR stained gel was scanned in a Chemidoc (ChemiDoc, Image Lab 4.1, Bio-Rad Laboratories, USA), using SYBR green filter with UV trans-illumination.

### Statistical Analysis

To compare the mean for more than two groups, we used one-way analysis of variance (ANOVA). It was followed by Tukey test for pairwise comparison. To compare the difference of mean between two groups, we used Student’s t-test. Values are expressed as mean ± SEM. A p-value less than 0.05 was considered statistically significant.
